# The challenges of today and tomorrow

**DOI:** 10.4103/0971-3026.38499

**Published:** 2008-02

**Authors:** Bhavin Jankharia

**Affiliations:** Editor-in-Chief, Indian Journal of Radiology and Imaging, Bhaveswar Vihar, 383 Sardar V. P. Road, Prathana Samaj, Mumbai - 400 004, India

As we enter the New Year, we are proud that we have been able to keep all the promises made earlier in 2007. This February 2008 issue should be with you in the first week of February. In fact, this issue was out on January 15 itself, a day before the start of the IRIA.

The IRIA, like the RSNA in the US, has now become a mega-event for radiology in India. Over 3-4 days, a few thousand delegates land up for the meeting, many hoping to brush up and add to their knowledge during the CMEs, though a good number, probably the majority, use the event mainly as a way of catching up with old friends from all over the country. It as much an academic event as it is a social networking one.

This issue does not have a special theme, but the focus is on PET/CT as well as diffusion tensor imaging. The case reports are predominantly focused on head and neck lesions as well as male and female genitourinary problems.

For me, the most exciting part of this issue has been the article on osteoclastoma that we have reproduced from the first volume of the IJR from 1947. It all started with a query from a medical business magazine called “Modern Medicare,” which wanted a copy of the first issue of the journal for their publication on the history of medicine in India. The first issue was not available, but we were able to locate the second one, thanks to Dr. Bharat Aggarwal. On going through the bound first volume, I was struck by how topical some of the articles still are. This is especially true of the osteoclastoma article, which has teaching value even today. It was a challenge to get the images to be of the same high quality as the other images in this issue, but we seem to have managed reasonably well. “From the Archives” will now be a regular feature in the IJRI and we will reproduce articles of interest in all subsequent issues.

This issue also has two articles on PET/CT. The review article by Dr. Amol Takalkar is a continuation of the first article that was published in the August 2007 issue on the physics and principles of PET/CT. This article reviews the clinical oncology applications. The next article in the May or August 2008 issue will review the non-oncology applications. As I have mentioned before, PET/CT is now as much a radiology modality as it is a nuclear medicine one and close collaboration and cooperation with our nuclear medicine colleagues has to be the norm as the modality becomes more and more widely used. The case report on the use of PET/CT for guided lung biopsy shows how this modality can influence the way we manage patients.

The May and August 2008 issues will focus on interventional radiology. Dr. Chander Mohan is the guest editor and most of the selected authors have already sent in their articles. However, those who would like to contribute original articles or pictorial essays on any interventional radiology topic are welcome to submit them at the earliest.

Dr. Jignesh Thakker is now the overall coordinator in the IRIA for all PC-PNDT related issues. Along with Dr. Bijal Jankharia, he has penned down a succinct list of “do's and don'ts.” At all meetings, we hear stories of how radiologists are being victimized by officials throughout the country. However, except for a couple of incidents, most of us have come to realize that the blame is usually the radiologists’, with either required forms not having been filled or boards not being in place or some other conditions not having been complied with. Please understand and remember this: “compliance with the provisions of the PC-PNDT Act is mandatory and a statutory requirement.” When officers come to inspect your premises for PC-PNDT compliance, please treat their presence with the same seriousness as you would an income-tax survey or raid and be polite and nice to the people who have come to the clinic.

The practice of radiology is changing significantly. The recent upsurge in the use of CT and MRI facilities has fueled a growth, which is in keeping with the growth of other industries in this country. We are slowly facing a manpower shortage: of technologists and radiologists. Machines are getting to be more and more expensive, corporate chains are getting into the act, hospitals are getting more aggressive, the referring doctors want a part of the action as well, and patients are becoming smarter and better informed. In this scenario, it is not enough for a radiologist to be good with his/her core knowledge of the subject; he/she also has to be good in finance with a better than working knowledge of managerial methods and legal issues, including compliance with statutory laws. We are hoping to address these topics in these pages in the months to come.

Feedback is important for us to understand the impact of what we do. Constructive criticism is also the best way of getting new ideas. Please feel free to get back to me and our team on editor@ijri.org. All feedback will be answered.

